# Meeting the HIV prevention needs of substance using young adults in the United States Virgin Islands

**DOI:** 10.1186/1742-4690-9-S1-P129

**Published:** 2012-05-25

**Authors:** Samuel MacMaster, John Wodarski

**Affiliations:** 1 University of Tennessee, Nashville Tn, USA

## Introduction

The need for comprehensive HIV prevention in the United States Virgin Islands is tremendous. HIV rates in the Carribbean region of the world is second only to sub-Saharan Africa, and the USVI has one of the highest rates in the United States. The primary mode of transmission in the USVI is heterosexual contact fueled by substance using risks. The presenation will provide an overview of a model program designed to increase HIV testing access and reduce HIV infections.

## Materials and methods

The project is a collaboration between local entities and the Univeristy of Tennessee and Norfolk State University. The program seeks to provide culturally appropriate early intervention, HIV testing, and substance use services to young adults at high risk for contracting HIV.

## Results

Over the first two years of the project over 160 individuals have participated in the project and have experienced statistically significant improvements in HIV risk behaviors and levels of substance use.

## Conclusions

The project serves as an example of culturally appropriate interventions for high risk young adult populations.

